# *Helicobacter pylori* antibiotic sensitivity pattern in dyspeptic patients in Kano, Nigeria

**DOI:** 10.4102/sajid.v34i1.125

**Published:** 2019-10-17

**Authors:** Ahmad K. Bello, Mohammad M. Borodo, Ahmad M. Yakasai, Abubakar D. Tukur

**Affiliations:** 1 Department of Internal Medicine, Faculty of Basic Clinical Sciences, Ahmadu Bello University, Ahmadu Bello University Teaching Hospital (ABATHU), Zaria, Nigeria; 2 Department of Internal Medicine, Faculty of Health Sciences, Bayero University Kano, Aminu Kano Teaching Hospital, Kano, Nigeria; 3 Department of Infectious Diseases, Faculty of Clinical Sciences, Public Health and Diagnostic Institute, College of Medical Sciences, North-West University, Kano, Nigeria; 4 Department of Microbiology, Faculty of Allied Health Science, Aminu Kano Teaching Hospital, Bayero University, Kano, Nigeria

**Keywords:** *H. pylori* Antibiotic Sensitivity, Kano – Nigeria, *H. pylori* Culture, Histology, Clarithromycin and Levofloxacin Sensitivity

## Abstract

**Background:**

Despite the high prevalence of *Helicobacter pylori* infection in Nigeria, in the North-West there are no studies on the antibiotic sensitivity pattern of this organism. This study aims to determine the antibiotic sensitivity pattern of this bacterium as well as bridge the gap in knowledge.

**Methods:**

The study was cross-sectional in design. Questionnaires were administered in dyspeptic patients to obtain the relevant data. Two sets of gastric biopsy specimens were taken during upper gastrointestinal (GI) endoscopy. One set was sent to the histopathology laboratory for assessment and *H. pylori* identification, while the other set for culture was minced and plated on Columbia blood agar media (Oxoid Ltd, England) incubated at 37°C in an anaerobic jar containing CampyGen (Oxoid Ltd) to provide the required micro-aerobic environment. The disc diffusion method was used in determining the sensitivity pattern of isolates. Pre-treatment and post-treatment stool samples were collected from each patient for a *H. pylori* faecal antigen test to assess eradication rate.

**Results:**

The sensitivity of *H. pylori* to amoxicillin was 9.2%, and 100% for both clarithromycin and levofloxacin. Tetracycline, metronidazole, cefuroxime, tinidazole and ciprofloxacin were 100% resitant. The prevalence of *H. pylori* at histology was 81.7%. Only 101 subjects had a positive *H. pylori* stool antigen test.

**Conclusion:**

This study showed a high amoxicillin resistance; however, there is high sensitivity (100%) to clarithromycin and levofloxacin. We recommended that levofloxacin be adopted in preference to amoxicillin as part of triple therapy in Nigeria.

## Introduction

*Helicobacter pylori* is the most important aetiologic risk factor of peptic ulcer disease (PUD) that infects human gastric tissue in half of the world’s population. There is a high prevalence of *H. pylori* in resource-poor regions of the world, particularly in Africa, which has the highest burden of the infection.^1,2,3^ With the high prevalence of *H. pylori* in our environment, not surprisingly resistance to some of the antibiotics used in its treatment is posing a huge challenge in eradicating the pathogen, leading to high recurrence of dyspeptic symptoms in patients who have completed *H. pylori* eradication therapy.

In a study conducted in Jos, North-central Nigeria, in 1999, *H. pylori* sensitivity to amoxicillin and clarithromycin was reported as 100% and 87.3%, respectively.^4^ However, recent studies in 2010 in South-West Nigeria^5^ reported 34% amoxicillin sensitivity, while in Cameroon, 55.3% clarithromycin sensitivity was reported,^6^ In the North-West zone of Nigeria (the study area), there is no data regarding the antibiotic sensitivity pattern of *H. pylori*. This study was therefore designed to determine the *in vitro* sensitivity pattern of *H. pylori* obtained from dyspeptic patients undergoing diagnostic oesophago-gastro-duodenoscopy (OGD) at Aminu Kano Teaching Hospital (AKTH), Kano, Nigeria. The study is also intended to bridge the gap and to contribute to the growing interest and knowledge in *H. pylori*, and to assist physicians in the choice of appropriate antibiotics for *H. pylori* eradication.

## Methods

The study was carried out in AKTH, a tertiary hospital in Kano, the most populous state in Nigeria, from August 2011 to July 2013. Aminu Kano Teaching Hospital is a public hospital in the North-West with a functioning endoscopy and endoscopic retrograde cholangiopancreatography facility (ERCP), and thus receives referrals from many states. The study was cross-sectional or interventional in design, and the ethical approval for the study was obtained from the research ethics committee of AKTH before the commencement of the study. Dyspeptic patients 18 years and above referred for upper gastrointestinal endoscopy, who consented to participate in the study, constituted the study population and were randomly recruited to meet the calculated sample size of 320 subjects. Any patients on antibiotics, proton pump inhibitors (PPI) or bismuth salts in the preceding 4 weeks, and those with medical conditions such as congestive cardiac failure and cardiac arrhythmias, were excluded from participating in the study. Written informed consent was sought and obtained from each patient before commencing the study and the procedure.

Patients were then booked for OGD and asked to fast overnight, for at least 8 h to the morning of the endoscopy. Each patient was given two sterile plastic containers to provide stool on the day of the endoscopy for pre-treatment *H. pylori* faecal antigen test, and the second stool sample collected 4 weeks post-eradication therapy to assess treatment outcome. At the endoscopy suite, the endoscopy procedure was explained to the patient, and then consent for endoscopy obtained. Thereafter, 10% xylocaine pharyngeal spray was administered to the patient’s pharynx. The patient was then placed on his or her left lateral position on the endoscopy couch, and a plastic dental guard was held firmly in the patient’s mouth by the assisting endoscopy nurse. A forward viewing fibre-optic Pentax FC-38 LW 2008 gastroscope was gently introduced under vision to examine the oesophagus, stomach and duodenum of the patients. During the procedure, two sets of four biopsy specimens, two each from the body of the stomach and antrum, were taken and the specimen for culture were immediately placed into screw top bottles with 1 mL of 0.9% sterile physiologic saline and transported within 1 h to the microbiology laboratory for *H. pylori* culture and antibiotic sensitivity. The other set fixed in 10% formaldehyde and sent to the histopathology laboratory for histologic assessment and identification of *H. pylori*.

The biopsy specimens for culture were minced by the attending microbiology scientist and then placed in a two plate media: the first medium being Columbia blood agar (Oxoid Ltd, Basingstoke, Hampshire, England) supplemented with 7% laked horse blood (Oxoid Ltd), and the second was Columbia blood agar (Oxoid Ltd) supplemented with 7% sheep blood (Oxoid Ltd). Dent’s supplement (Oxoid Ltd), containing vancomycin 5.0 mg, trimethoprim lactate 2.5 mg, cefsulodin 2.5 mg and amphotericin B 2.5 mg, was also added to the two plates. Both plates were then incubated at 37 °C in an anaerobic jar containing CampyGen (gas generating kits) (Oxoid Ltd) to provide the micro-aerobic environment required for growth of the organism. The plates were checked daily over 3–5 days for growth. *Helicobacter pylori* isolates were identified by using Gram stain features, urease, catalase and oxidase activities. Identified *H. pylori* colonies were then sub-cultured on Columbia blood agar supplemented with 7% sheep blood to get a heavy growth.

### Antimicrobial susceptibility

Eight antimicrobial agents were tested against each *H. pylori* isolate using the antibiotic test discs (amoxicillin, levofloxacin clarithromycin and tetracycline) (Oxoid Ltd). Other drugs are metronidazole, ciprofloxacin, cefuroxime and tinidazole. Inoculum from the subculture was made and streaked onto Brucella Agar containing 7% sheep blood. The antibiotic strips were added and then the plates were incubated at 37 °C in a micro-aerobic environment provided by the CampyGen system (Oxoid Ltd) for another 3–4 days. The antibiotics’ susceptibility (S) and resistance (R) were recorded for each of the antibiotics previously listed.

In the histopathology laboratory, the paraffin-embedded tissue blocks were sectioned at 4 µm thickness and stained with the routine H and E stain for morphology, while modified Giemsa and Warthin-Starry stains were used for the identification of *H. pylori*. Sections found to show spiral bacteria in the mucosal layer or on the surface of the gastric epithelial cells were considered positive for *H. pylori* as assessed independently by two histopathologists.

### Helicobacter pylori faecal antigen test

A stool sample was collected from each patient on the day of endoscopy to assess for *H. pylori* faecal antigen using *H. pylori* Antigen RapiCard Instatest card (Cortez Diagnostics Inc., Calabasas, CA, United States [US]). If found to be positive, amoxicillin 1 g bid and clarithromycin 500 mg bid along with rabeprazole 20 mg od were prescribed to the patients to take for 14 days, and they were instructed to bring a second stool sample 4 weeks after completing the medications (post-treatment) to assess the eradication or otherwise. The faecal antigen tests were performed on fresh stool samples according to the manufacturer’s direction as described below. The required numbers of sample diluent vials were labelled with the sample identity number and the cap removed. The applicator stick, which was attached to the cap of the stool specimen bottle, was used to transfer a small piece of formed stool into the sample bottle containing specimen preparation buffer, whereas a liquid or semi-solid stool sample (100 mL of sample) was added to the vial using a disposable pipette. The cap was replaced and the mixtures homogenised for 15 seconds by manually shaking vigorously. With the cassette removed from the sealed foil pouch, the sample bottle tip was snapped off and three drops of diluted sample was added to the well. The result was read within 10–15 minutes.

### Statistical analysis

Data obtained were analysed using Statistical Package for Social Sciences, version 16.0 (SPSS Inc., 2009, Chicago, IL, US). Quantitative variables were summarised using range, means and standard deviation, while qualitative variables were summarised using ratios, proportions and percentages. A value of *p* < 0.05 was regarded as statistically significant. Determinants and predictors were explored using univariate and multivariate analyses with logistic regression.

### Ethical considerations

Ethical approval to conduct the research on antibiotic sensitivity pattern in dyspeptic patients in Kano, Nigeria, was obtained from the Ethical Committee of the Aminu Kano Teaching Hospital, Kano, Nigeria (clearance number: AKTH/MAC/SUB/12A/P3/IV/608).

## Results

Table 1 shows 136 (44.4%) participants were males, while 170 (55.6%) were females, with a male:female ratio of 1:1.3. The ages of the subjects ranged from 18 to 84 years, with a mean age of 41.2 ± 15.3 years.

**TABLE 1:** The prevalence of *Helicobacter pylori* according to gender distribution of the study subjects.

Sex	Subjects	*H. pylori* positives	Prevalence rate (%)
Males	136	112	82.4
Females	170	138	81.2
Total	306	250	81.7

*H. pylori, Helicobacter pylori.*

Of the 320 samples sent to the histology laboratory for analysis, 14 (4.4%) were lost during processing. The remaining 306 samples were examined by the pathologist, out of which 250 (81.7%) were positive for *H. pylori*, while 56 (18.3%) were negative. This gave *H. pylori* a prevalence of 81.7% at histology.

### Helicobacter pylori culture

Out of the 320 samples cultured, 109 grew *H. pylori*, while the remaining 211 were negative, as seen in Table 2. Of the 109 subjects who were *H. pylori* culture positive, 55 (50.5 %) were males, while 54 (49.5 %) were females, with male:female ratio of 1:1.

**TABLE 2:** Endoscopic finding with distribution of *Helicobacter pylori* at culture.

*Helicobacter pylori* culture					
Endoscopic finding	Positive		Negative		*p*
	*N*	%	*N*	%	
Normal	24	49.0	25	51.0	0.56
Duodenal ulcer	6	66.7	3	33.3	0.31
Gastric ulcer	11	36.7	19	63.3	0.90
Gastric cancer	1	12.5	7	87.5	0.43
Gastritis	42	29.8	98	70.2	0.90
Duodenitis	23	45.1	28	54.9	0.69
Oesophagitis	2	8.7	21	91.3	0.20
Oesophageal cancer	0	0.0	3	100	0.27
Hiatus hernia	0	0.0	2	100	0.36
Candida oesophagitis	0	0.0	4	100	0.21
**Total**	**109**	**34.1**	**211**	**65.9**	**-**

### *Helicobacter pylori* antibiogram

As depicted in Table 3, the sensitivity of the 109 *H. pylori* isolates cultured is as follows.

**TABLE 3:** *Helicobacter pylori* antibiogram.

Antibiotic tested	Susceptible		Resistant	
	*N*	%	*N*	%
Amoxicillin	10	9.2	99	90.8
Metronidazole	0	0.0	109	100.0
Tetracycline	0	0.0	109	100.0
Tinidazole	0	0.0	109	100.0
Ciprofloxacin	0	0.0	109	100.0
Clarithromycin	109	100.0	0	0.0
Cefuroxime	0	0.0	109	100.0
Levofloxacin	109	100.0	0	0.0

All 109 (100%) *H. pylori* isolates were sensitive to clarithromycin and levofloxacin, but only 10 (9.2%) isolates were sensitive to amoxicillin. None of the isolates were sensitive to metronidazole, tetracycline, tinidazole, ciprofloxacin and cefuroxime.

## Discussion

**FIGURE 1:**
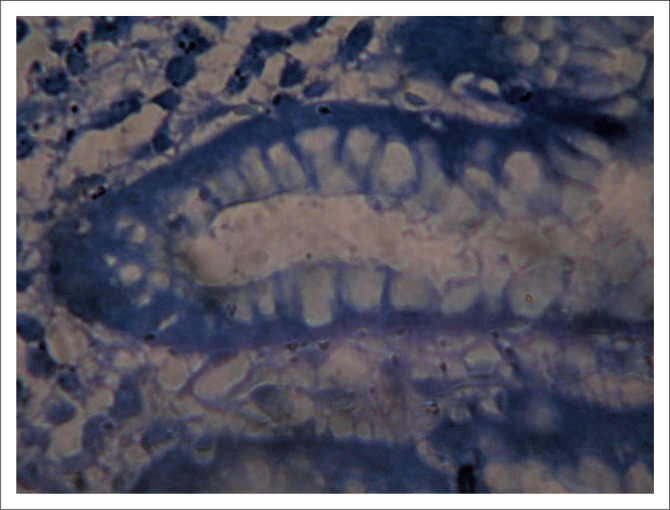
*Helicobacter pylori* in gastric biopsy at histology.

The prevalence of *H. pylori* in this study was 81.7% using histology, as seen in Table 1, which is the gold standard for the detection of *H. pylori* in our environment (Figure 1). This prevalence is consistent with *H. pylori* prevalence rates as reported in other Nigerian studies. An earlier study in 2009 from the same centre by Bashir and Ali^7^ reported 81% prevalence. In another study from Gombe^8^ *H. pylori* prevalence was found to be 77.1%, while Ndububa et al.^9^ in Ile-Ife reported prevalence rates of 73%. Nigerian prevalence rates are similar to those reported in studies in South Africa^10^ and Kenya^11^ which reported, respectively, rates of 66% and 94%. Woodward et al.^12^ in a study in Glasgow, United Kingdom, reported a prevalence of 66% which they noted was more typical of prevalence in developing countries and thus concluded that the high degree of social deprivation in Glasgow at that time was the explanation for the high prevalence of *H. pylori* found in the study. In our study, as shown in Table 1, the prevalence of *H. pylori* infection is higher in males (82.4%) than females (81.6%). Univariate analysis on demographic factors in this study showed that there is an increased prevalence of *H. pylori* in male subjects, which is statistically significant (*p* < 0.0041) when compared with that of females. Similarly, Omosor et al.^13^ revealed that the prevalence of *H. pylori* infection was also higher in males (55%) than females (51.4%). Woodward et al. also reported a higher prevalence of *H. pylori* in men than in women.^12^ Ford and Axon observed that male gender is a risk factor for *H. pylori* infection.^14^ The distribution of *H. pylori* at histology in the various endoscopic findings can be seen in Table 4.

**TABLE 4:** Endoscopic finding with distribution of *Helicobacter pylori* at histology.

*Helicobacter pylori* histology							
Endoscopic finding	Positive		Negative		Total		*P*
	*N*	%	*N*	%	*N*	%	
Normal	41	85.4	7	14.6	48	15.7	0.85
Duodenal ulcer	8	88.9	1	11.1	9	2.9	0.69
Gastric ulcer	25	83.3	5	16.7	30	9.8	0.97
Gastric cancer	2	40.0	3	60.0	5	1.6	0.00
Gastritis	121	89.6	14	11.0	135	44.2	0.03
Oesophagitis	18	78.3	5	21.7	23	7.5	0.58
Duodenitis	42	85.7	7	14.3	49	16.0	0.00
Hiatus hernia	0	0	1	100.0	1	0.3	0.60
Oesophageal cancer	0	0	3	100.0	3	1.0	0.64
Candida oesophagitis	1	33.3	2	66.7	3	1.0	0.86
**Total**	**258**	**84.3**	**48**	**15.7**	**306**	**100**	**-**

### *Helicobacter pylori* antibiotic profile

This study revealed an antimicrobial susceptibility rate of only 9.2% for amoxicillin, an antibiotic that is commonly used in triple therapy for *H. pylori* eradication in Nigeria (Table 3). Jaka et al. in a systematic review and meta-analysis involving 26 articles reported a 72.6% *H. pylori* resistance to amoxicillin,^15^ and concluded that prevalence of metronidazole, clarithromycin and amoxicillin resistance is high in the developing world including Africa, which, they said, could impair the first-line triple therapy of *H. pylori* infection. An earlier study in Western Nigeria^5^ reported 34% amoxicillin sensitivity, while in 2007 Aboderin et al. reported 100% amoxicillin resistance in their *H. pylori* isolates.^5^ Ndip et al. in Cameroon reported 14.4%^6^ and Wu et al. in a study in China^16^ reported a rate of 28.1%. However, Ani et al. in North-Central Nigeria,^4^ almost two decades earlier, had reported 100% susceptibility to amoxicillin. This study coming from same part of the country highlighted the likely acquisition of resistance overtime. Other studies from Malaysia, Saudi Arabia and Lebanon reported 100%, 99% and 100% sensitivity to amoxicillin, respectively.^17,18,19^ These differences in antibiotic susceptibility in the different countries could be attributed to the differences in local antibiotic prescription practices and usage in the various countries at the time of the studies. In Nigeria, drug regulations are lacking and the few laws enacted are not enforced, thus antibiotics and other drugs are sold over the counter leading to their abuse, thus encouraging the development of resistance in the individuals later. In addition, there is widespread proliferation of cheap and substandard drugs including antibiotics in the country which encourages the development of resistance not only to *H. pylori* but to other infections as well. Studies have shown that in Africa amoxicillin and ampicillin are the most abused antibiotics in both rural and urban areas because they are cheaply available in oral formulations.^20^

**FIGURE 2:**
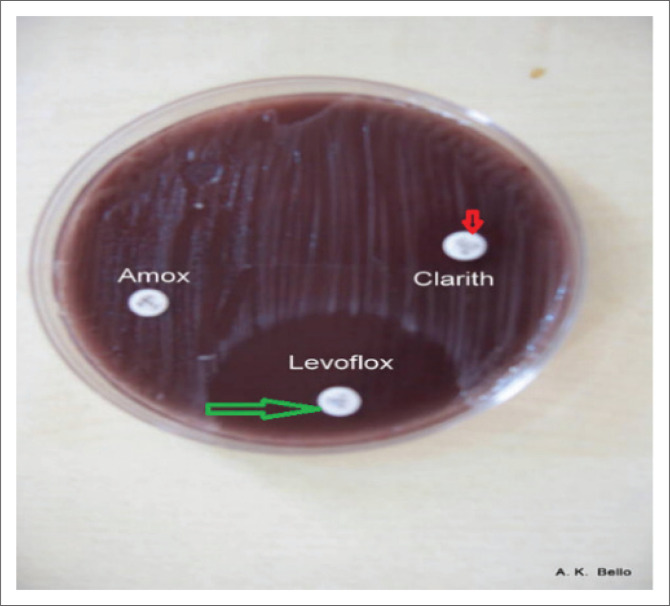
*Helicobacter pylori* growth inhibited by levofloxacin (green) and clarithromycin (red) above.

We recorded 100% sensitivity to clarithromycin (Figure 2) in this study, which was similar to that of Kimanga et al.^11^ in Kenya, Rizwan et al. in Saudi^18^ and Sharara et al.^19^ in Beirut, who reported 100%, 96% and 96% clarithromycin susceptibility, respectively. Clarithromycin resistance the world over was reported as low, ranging from 0% to 45%.^18,21,22^ Studies from Malaysia^17^ reported 97.9% and in the United States^23,24^ 93.9% sensitivity. However, Kumala et al.^25^ in Indonesia and another study from Cameroon^6^ reported lower rates of clarithromycin susceptibility of 72.2% and 55.3%. A major contrast to the findings in our study and the others noted above is a study in Western Nigeria,^5^ which reported that 100% of their *H. pylori* isolates were resistant to clarithromycin. We are not aware of any other study globally that has reported 100% clarithromycin resistance; therefore, the reason for this marked difference is not clear because clarithromycin resistance is very low and as such it is used widely in the treatment of *H. pylori* infections in most parts of the world.

All 109 (100%) isolates in our study were susceptible to levofloxacin (Figure 2). To our knowledge, there is no study that has tested levofloxacin sensitivity in Nigeria. Norazah et al.^17^ in Malaysia reported 99% sensitivity to levofloxacin, while a systemic review^26^ showed a susceptibility rate of 88.4% in Asia, 75.9% in Europe and 0% in Africa. Levofloxacin can therefore be a suitable substitute to amoxicillin in the triple therapy used for *H. pylori* eradication in Nigeria.

**FIGURE 3:**
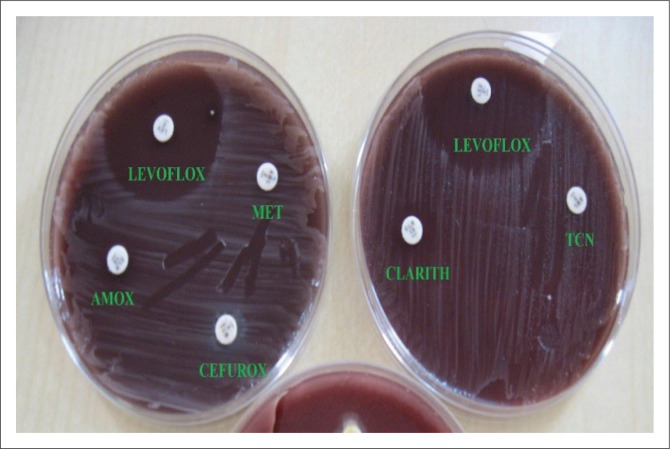
*Helicobacter pylori* growth un-inhibited by tetracycline, metronidazole or cefuroxime.

We found 100% resistance of our isolates to ciprofloxacin as seen in Figure 3; however, a study from Ile-Ife^5^ in Western Nigeria, a decade earlier, showed 15.6% ciprofloxacin resistance, and the resistance rate in Jakarta was 6.9%.^25^ The reason for this difference is not apparent; however, different antibiotic usage and abuse in the two regions as well as the time of the studies could be responsible for this difference. In Nigeria, there is tremendous pressure on the use of ciprofloxacin by general practitioners for the treatment of typhoid fever, the diagnosis that is often erroneous. Moreover, ciprofloxacin is cheap and readily available in both rural and urban areas, and thus abused by individuals and general practitioners. On the contrary, levofloxacin is new and expensive and as such not subjected to abuse, which may explain the noted susceptibility seen in our study. To our knowledge, there is no other *H. pylori* study on levofloxacin in Nigeria.

The high resistance rate of 100% observed for metronidazole and tinidazole (Figure 3) is comparable to numerous studies from many developing countries that have reported similar results to our findings. Two separate studies done in Western Nigeria reported 95% and 100% resistance to metronidazole.^5,11^ Studies in Cameroon, Indonesia and China reported 93.2%, 63.9% and 100% resistance to metronidazole, respectively.^6,25,27^ These findings could be attributed to the poor drug regulation in Africa and other developing countries leading to high antibiotic abuse that encourages development of resistance. Metronidazole resistance has been reported in 10% – 50% of patients in developed countries,^28^ whereas in developing countries virtually all strains of *H. pylori* have been found to be resistant to metronidazole.^29^ The high prevalence of metronidazole resistance in developing countries like Nigeria may also be related to the frequent use of imidazole derivatives for the treatment of gastrointestinal and gynaecological infections often at substandard doses for the relief of diarrhoeal disease which are of frequent occurrence. Osato et al.^30^ reported that metronidazole resistance was seen more in females (63%) than their male (35%) counterparts. From other studies, metronidazole resistance of *H. pylori* has been shown to be because of the mutational inactivation of the nitroreductase gene (rdxA gene), which encodes nitro-reductase, an enzyme gene, which encodes nitro-reductase, an enzyme that reduces metronidazole to an active form.^29^

There was 100% *H. pylori* resistance to tetracycline in this study as seen in Figure 3, which was similar to two other studies by Oladiipo et al., and Smith et al. in Western Nigeria^5,31^ which all reported 100% tetracycline resistance. Tetracycline is not currently used for *H. pylori* eradication in Nigeria because of its high resistance profile. However, in China and Malaysia^16,17^ studies reported resistance rates of 1.2% and 0% to tetracycline, and Sharara in Beirut^19^ also reported 2% tetracycline resistance.

In this study, 100% of *H. pylori* isolates were resistant to cefuroxime (Figure 3); in contrast, a study in China^27^ reported resistance of only 1.1%. Differences in the antibiotic usage and abuse in the two continents may be responsible for this contrast.

### Rapid *Helicobacter pylori* faecal antigen test

**FIGURE 4:**
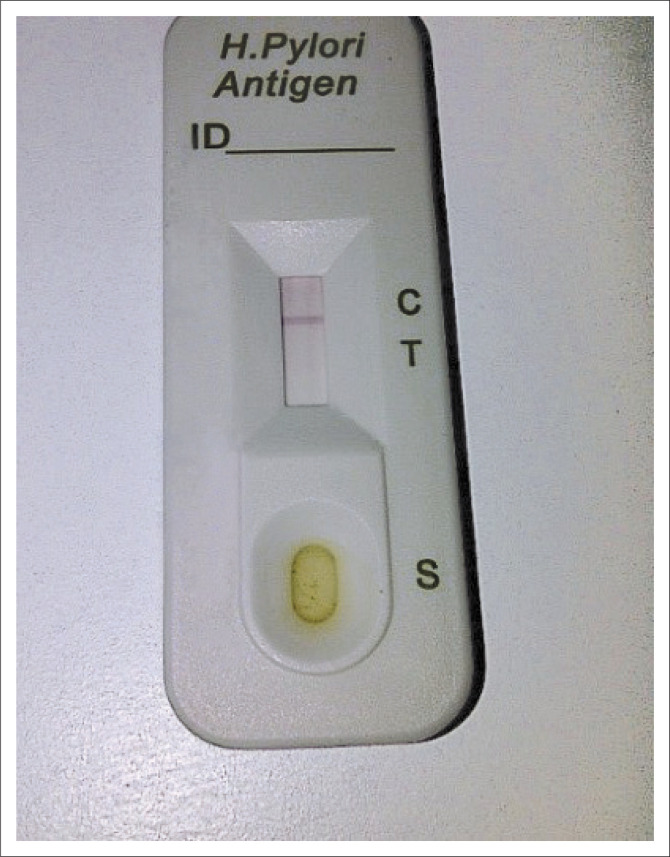
Positive *Helicobacter pylori* faecal antigen test (presence of horizontal line on C only).

**FIGURE 5:**
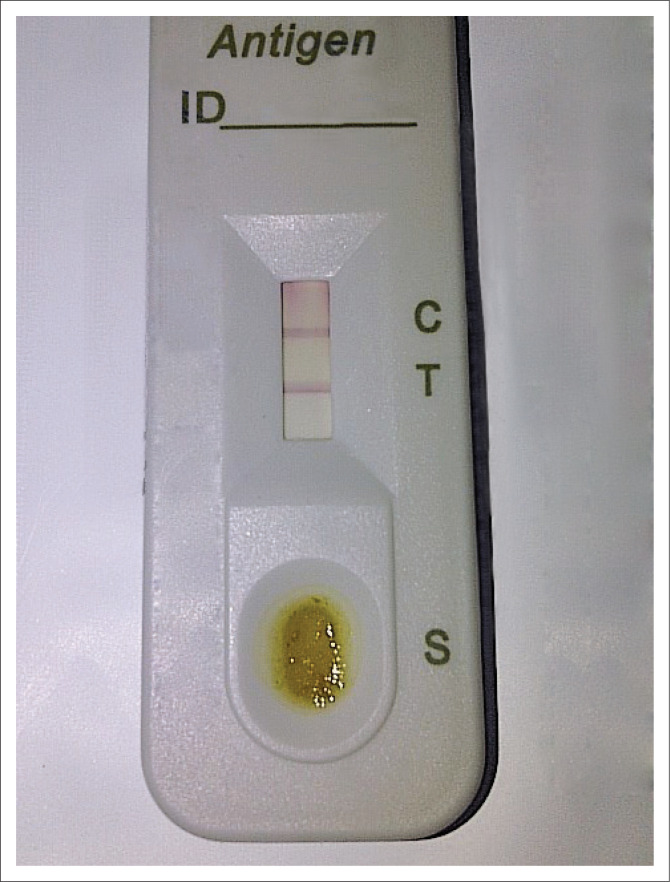
Positive *Helicobacter pylori* faecal antigen test (presence of horizontal lines on C & T) above.

One hundred and one subjects (34.0%) were *H. pylori* faecal antigen test positive pre-treatment, as shown in Table 5 and Figure 4. Of the 101 initially positive, 9 patients defaulted, while 92 came back for a post-treatment eradication test (rapid *H. pylori* stool antigen test [*Hp* SAT]). Of these, 12 (13.0%) were still positive post-treatment (see Figure 5 and Table 6). Smith et al.^31^ reported that 53% of their subjects were still positive post-treatment. The prevalence of *H. pylori* using the *Hp* SAT in this study was 31.6%, which is similar to the 36.7% reported by Stella et al. in Lagos-Nigeria.^32^ Currently available eradication regimens for *H. pylori* are triple drug combination regimens comprising a PPI and two antibiotic drugs, and eradication rates of 70% – 90% are obtained using these regimens.^21^ The eradication rate of 86.9% achieved by our study agrees with the above finding. The eradication rates of *H. pylori* have been lower than 80% as a result of increased resistance to *H. pylori*.^33^ Gisbert et al. in a systematic review of 89 studies evaluated the *H. pylori* stool antigen test and reported a sensitivity of 96% with a specificity of 97%.^34^ The eradication rate may have been lower if re-endoscopy with *H. pylori* culture and histology was done. We probably achieved this high eradication level despite over 90% amoxicillin resistance because of the fact that proton pumps inhibitors (PPI) in themselves inhibit *H. pylori*. This property of PPI and the 100% susceptibility to clarithromycin of our isolates would possibly explain this high eradication rate we recorded in spite of the amoxicillin resistance. It could also be explained that the free drugs (PPI, amoxicillin and clarithromycin) provided by the researcher for the required duration of treatment have encouraged patients’ compliance and thus led to the achieved high eradication rate noted. The apparent high eradication rate mainly from clarithromycin effect without the support of a second antibiotic should not be taken for granted – as guidelines generally recommend – for this may expose this robust antibiotic (clarithromycin) in the future to the development of resistance by the *H. pylori* bacteria.

**TABLE 5:** Pre-treatment *Helicobacter pylori* faecal antigen test.

Hp stool antigen	*Helicobacter pylori* faecal antigen test	
	*n*	%
Positive	101	34.0
Negative	196	66.0
**Total**	**297**	**100.0**

Hp, *Helicobacter pylori.*

**TABLE 6:** Post-treatment *Helicobacter pylori* faecal antigen test.

Hp stool antigen	*Helicobacter pylori* faecal antigen test	
	*n*	%
Positive	12	13.0
Negative	80	87.0
**Total**	**92** †	**100.0**

Hp, *Helicobacter pylori.*

† , Nine patients out of the 101 positives were lost to follow-up during the post-treatment faecal antigen test.

### Study limitations

The high cost of endoscopy and the discomfort of the procedure prevented patients from agreeing to return for repeat endoscopy and biopsy (only 10 out of 320 patients agreed to return for post-treatment repeat endoscopy). We thus had to resort to *in vitro* testing using the *H. pylori* faecal antigen test which correlated well with the culture result (see Table 7) in place of the initially intended *in vivo* testing of the antibiotic sensitivity using a culture to determine the eradication rate.

**TABLE 7:** *Helicobacter pylori* positivity using the three deferent methods.

Endoscopy findings	*n*	Histology	Culture	FAT 1	FAT 2
Normal	49	41	24	8	1
Duodenal ulcer	9	8	6	7	0
Gastric ulcer	30	25	11	16	2
Gastric tumour	8	2	1	0	0
Gastritis	141	121	42	51	8
Duodenitis	51	42	23	18	1
Oesophagitis	23	18	2	1	0
Oesophageal tumour	3	0	0	0	0
Hiatus hernia	2	0	0	0	0
Oesophageal candida	4	0	0	0	0
**Total**	**320**	**257**	**109**	**101**	**12**

FAT 1, pre-treatment faecal antigen test positivity; FAT 2, post-treatment faecal antigen test positivity.

## Conclusion

This study showed that *H. pylori* isolates in Kano, Nigeria, are resistant to metronidazole or tinidazole, tetracycline and amoxicillin, which are among the antibiotics used in *H. pylori* eradication therapy in the country, but are sensitive to clarithromycin and levofloxacin. Therefore, this finding, though limited to only one region of the country, may provide part of the basis for the need to review the current *H. pylori* antibiotic combination therapy currently in use in Nigeria.
